# RefSeq-corrected transcriptomic and RNA-interaction integration maps medium-associated vancomycin response rewiring in *Staphylococcus aureus*

**DOI:** 10.3389/fmicb.2026.1930249

**Published:** 2026-09-14

**Authors:** Zhangfu Li, Huanmei Liu, Baicheng Yang, Shiqing Liu, Zhenfeng Li, Shengji Yu

**Affiliations:** 1 Department of Orthopaedics, National Cancer Center/National Clinical Research Center for Cancer/Cancer Hospital, Chinese Academy of Medical Sciences and Peking Union Medical College, Beijing, China; 2 Department of Hospital Infection Management, National Cancer Center/National Clinical Research Center for Cancer/Cancer Hospital, Chinese Academy of Medical Sciences and Peking Union Medical College, Beijing, China

**Keywords:** public transcriptomics, RefSeq annotation, response rewiring, RNA–RNA interaction, *Staphylococcus aureus*, vancomycin

## Abstract

**Background:**

*Staphylococcus aureus* is a major cause of bone, joint and orthopaedic implant-associated infections, providing clinical motivation for understanding adaptive antibiotic-response states. However, the present study is a secondary analysis of public *in vitro* transcriptomic and RNA-interaction datasets rather than an infection-model or clinical-specimen study. Vancomycin remains important for serious MRSA infections, but adaptive responses to vancomycin may vary across environmental and evolutionary states. Here, we used a framework integrating RefSeq-harmonized transcriptomics and RNA–RNA interaction evidence to examine vancomycin response rewiring.

**Materials and methods:**

In this secondary analysis, we performed a processed-expression reanalysis of public *S. aureus* transcriptomic and RNA-interaction datasets, using GSE149213 as the discovery dataset for medium-stratified vancomycin response modeling across cation-adjusted Mueller-Hinton broth (CA-MHB) and Roswell Park Memorial Institute medium supplemented with 10% lysogeny broth (RPMI + 10% LB) in wild-type, media-adapted control and analytically pooled vancomycin-evolved lineages/populations. Candidate identities were harmonized using USA300HOU RefSeq locus-tag annotations, clean GSE254530 was used for conservative cross-dataset assessment, and published RNase III-CLASH data were used to evaluate candidate-associated RNA-interaction evidence.

**Results:**

In the exploratory GSE149213 processed-expression contrast analysis, analytically pooled vancomycin-evolved lineages/populations retained broad acute responses, with the strongest response-rewiring burden corresponding to the EVO-WT response shift in RPMI + 10% LB; EVO-MAC response shifts were much more limited. RefSeq locus-tag harmonization retained a predefined nine-locus candidate panel for evidence ranking. Conservative GSE254530 assessment provided strict cross-dataset differential-expression support for pyrR only, while glnA was uniquely mapped but not differentially expressed and the remaining seven candidates lacked conservative clean-feature mappings. RNase III-CLASH integration highlighted carbamoyl-P synthase, dihydroorotase and glnA as top-ranked candidate-associated CLASH hotspots. Module-level processed-expression analysis further localized evolved response shifts to RPMI-associated redox/stress, cell-wall/envelope and proteolysis/stress/virulence programs, with translation/ribosome showing a contrasting negative trend.

**Conclusion:**

This study provides a RefSeq-corrected, evidence-ranked processed-expression map of vancomycin response rewiring in *S. aureus*. Although the findings remain hypothesis-generating rather than causal, they define prioritized candidate loci, candidate-associated RNA-interaction edges and functional modules for future experimental testing in matched *in vitro* systems and biologically relevant infection models, including bone- and implant-associated models.

## 1 Introduction

*Staphylococcus aureus* is a major cause of bone, joint and orthopaedic implant-associated infections, including osteomyelitis, fracture-related infection and prosthetic joint infection (Masters et al., 2022; Patel, 2023). These infections are difficult to eradicate because bacteria can persist within biofilms, poorly vascularized bone-associated tissue, intracellular niches and inflammatory microenvironments, while treatment often requires prolonged antimicrobial therapy combined with surgical source control (Gatti et al., 2022; Gimza and Cassat, 2021). More broadly, antimicrobial-resistant *S. aureus* remains a leading bacterial threat, with recent global burden analyses placing *S. aureus* and methicillin-resistant *S. aureus* among the major contributors to infection-associated and antimicrobial-resistance-associated mortality (GBD 2019 Antimicrobial Resistance Collaborators, 2022, 2024). Vancomycin continues to be a central therapeutic option for serious MRSA infections, as reflected in contemporary consensus guidance for vancomycin use and monitoring (Rybak et al., 2020). However, vancomycin efficacy is challenged by reduced susceptibility, intermediate-resistance phenotypes and adaptive trajectories that can emerge without a simple one-gene explanation (Fait et al., 2024; Machado et al., 2021). These observations point to a broader biological problem: vancomycin defence in *S. aureus* is shaped not only by resistance endpoints, but also by how bacterial transcriptional response programs are remodelled across environmental and evolutionary states.

Although the discovery datasets analysed here were not generated from orthopaedic infection specimens, bone and implant-associated infection provides a clinically relevant motivation for asking how environmental context may reshape antibiotic-response states. This clinical framing is therefore used as a translational context rather than as direct evidence from infection models. Accordingly, the central question addressed here is a foundational one: whether *S. aureus* vancomycin-response programs in public *in vitro* datasets behave as fixed strain properties or as condition-dependent states that can be remodelled by growth environment and adaptation.

Mechanistic studies support this adaptive view of vancomycin defence. Reduced vancomycin susceptibility has been linked to cell-envelope remodeling and cell-wall thickening, but recent work also emphasizes that these phenotypes arise through heterogeneous genetic and regulatory routes rather than a single conserved pathway (Cui et al., 2021; Malachowa et al., 2024). Regulatory control of the cell-wall stress system further illustrates this complexity: for example, AgrA has been shown to directly regulate the vraSR promoter, connecting virulence regulation with a core vancomycin-responsive stress pathway (Liu et al., 2024). Because these mechanisms can vary with strain background, growth condition and evolutionary history, transcriptomic analysis needs to separate basal adaptation from the acute vancomycin response and from evolved-state changes in that response (Aguglia et al., 2023). Medium-stratified response-rewiring analysis therefore provides a way to distinguish genes that simply respond to vancomycin from genes whose inducible response is remodelled after media adaptation or vancomycin evolution.

Post-transcriptional regulation adds a second layer to this problem. Bacterial small RNAs and RNA–RNA interactions can coordinate stress responses and antibiotic-associated phenotypes, but transcript abundance alone does not reveal whether a vancomycin-responsive gene is embedded in an experimentally supported RNA regulatory network (Mediati et al., 2021). In *S. aureus*, RNase III-CLASH has recently expanded this view by mapping RNA–RNA interaction networks in MRSA and by identifying a regulatory mRNA 3′UTR required for intermediate vancomycin resistance (McKellar et al., 2022; Mediati et al., 2022). More recent interactome work has further linked a vancomycin-responsive small RNA, RsaOI, to targets in vancomycin-tolerant *S. aureus* (Wu et al., 2024). These studies suggest that integrating transcriptomic response rewiring with RNA-interaction evidence could help distinguish broad stress-response changes from candidates with stronger regulatory-network support.

A practical barrier to such integration is that public transcriptomic and interactome datasets are often interpreted through different layers of genome annotation. Gene symbols, product names and display labels can change across annotation releases, whereas curated RefSeq records and prokaryotic genome annotation pipelines provide locus-level anchors for more reproducible cross-dataset interpretation (Goldfarb et al., 2025; Haft et al., 2024). Without this step, candidate genes nominated from a discovery transcriptome may be difficult to compare with independent result tables or RNA-interaction resources, and apparent biological support can be inflated by label mismatch rather than true convergent evidence (Chorlton, 2024). For vancomycin-response studies in *S. aureus*, this creates a need for a conservative framework that first harmonizes candidates at the RefSeq locus-tag level, then evaluates whether those loci show response rewiring, conservative cross-dataset support and retained RNA-interaction evidence.

Here, we re-analysed public *S. aureus* vancomycin-response datasets to build a RefSeq-corrected framework for candidate prioritization across transcriptional response, conservative cross-dataset assessment and RNA-interaction evidence. We first modelled medium-stratified vancomycin response rewiring in GSE149213, treating the EVO group as analytically pooled evolved lineages/populations, then audited candidate identities using RefSeq locus-tag anchors and evaluated conservative support from an independent vancomycin-response RNA-seq dataset. We next integrated retained high-confidence RNase III-CLASH interactions to identify candidates embedded in experimentally supported RNA-interaction evidence, and summarized response changes at both candidate and functional-module levels. This analysis does not claim direct experimental validation of candidate mechanisms; rather, it provides a conservative, annotation-aware prioritization framework for interpreting vancomycin-associated RNA networks in *S. aureus*.

## 2 Materials and methods

### 2.1 Public datasets and study design

The GSE149213 dataset was used as the primary discovery dataset for medium-stratified vancomycin response analysis. The design included WT *S. aureus*, media-adapted control (MAC) populations and vancomycin-evolved (EVO) samples grown under two culture conditions, cation-adjusted Mueller-Hinton broth (CA-MHB) and Roswell Park Memorial Institute medium supplemented with 10% lysogeny broth (RPMI + 10% LB), with matched untreated and acute vancomycin-treated samples. The processed dataset contained 48 RNA-seq samples. Within each medium and treatment condition, WT and MAC each included two biological replicate samples. The EVO group contained eight samples per medium and treatment condition, corresponding to four evolved strain/lineage labels with two samples each: SVAM_A3, SVAM_A9, SVAM_A10 and SVAM_A12 in CA-MHB, and SVAR_A2, SVAR_A3, SVAR_A4 and SVAR_A5 in RPMI + 10% LB. These EVO samples were analyzed as an analytically pooled evolved-lineage/population group, not as ordinary replicate measurements of a single evolved clone. Full sample annotations are provided in Supplementary Table 2.

GSE149213 was used for sample-level transcriptomic structure, exploratory genome-wide vancomycin-response modeling, candidate response trajectories and module-level response-rewiring analysis. An independent clean vancomycin-response RNA-seq reference, GSE254530, was used for conservative cross-dataset assessment of RefSeq-corrected candidates. This assessment was restricted to candidates that could be conservatively remapped to the clean GSE254530 annotation and evaluated in the VAN30 DESeq2 differential-expression table.

RNA-interaction evidence was obtained from the published *S. aureus* RNase III-CLASH interactome dataset generated by Mediati et al. (GSE158830). This dataset was not used to infer a complete RNA-interaction network in the present study. Instead, it was used to identify retained interpretable high-confidence CLASH edges involving the RefSeq-corrected prioritized candidates. Together, these datasets supported a workflow linking genome-wide vancomycin response, RefSeq locus-tag harmonization, conservative cross-dataset support and candidate-associated RNA-interaction evidence.

### 2.2 GSE149213 expression matrix and sample-level structure

GSE149213 analyses were performed as a processed-expression reanalysis using the available GEO processed TPM expression matrix. Expression values were transformed as log2(TPM + 1) before downstream analysis. The retained processed matrix contained 2,888 genes across 48 samples. Unless otherwise stated, downstream transcriptomic analyses used this log2(TPM + 1) matrix. Raw read-count matrices were not available among the retrieved processed GEO files, and no raw-count DESeq2, edgeR, limma-voom or raw-count sensitivity workflow was performed. Although the GEO processed file name contains “deseq2-tpm,” the present analysis used the file as a processed TPM expression matrix and did not treat it as a raw-count DESeq2 input.

Sample-level transcriptomic structure was assessed using pairwise Pearson correlation between samples calculated from the log2(TPM + 1) expression matrix. The resulting sample-correlation matrix was visualized with hierarchical clustering and annotated by strain, evolutionary state, treatment and culture medium. Principal-component analysis was used as an additional quality-control and exploratory analysis, using the 1,000 most variable genes from the same transformed expression matrix. The first two principal components explained 33.1 and 15.8% of the variance, respectively.

For candidate-level response trajectories, acute vancomycin response was summarized from replicate-level expression values. For each RefSeq-corrected candidate, medium and evolutionary state, response was calculated as the mean log2(TPM + 1) expression in vancomycin-treated samples minus the mean log2(TPM + 1) expression in matched untreated samples. Thus, candidate trajectory values represent observed group-level response summaries rather than model-derived limma coefficients.

### 2.3 Genome-wide response and response-rewiring analysis

Genome-wide vancomycin responses and response-rewiring contrasts were estimated from the log2(TPM + 1) expression matrix using limma as an exploratory processed-expression analysis. Because sample-level structure and response magnitude differed by culture condition, CA-MHB and RPMI + 10% LB samples were first analysed separately. Within each medium, a no-intercept design matrix was constructed using the combined evolutionary state and treatment group, thereby modeling untreated and vancomycin-treated samples for WT, MAC and the analytically pooled EVO group. Gene-wise linear models were fitted with limma, followed by empirical Bayes moderation. False discovery rates were calculated using the Benjamini-Hochberg procedure and are reported as FDR-adjusted statistics from processed-expression limma contrasts, not as raw-count RNA-seq differential-expression calls. To directly test whether response shifts differed between media, we also fitted a combined-medium limma model containing medium, evolutionary state and treatment groups, and estimated medium-interaction contrasts comparing the RPMI + 10% LB response-shift coefficient with the corresponding CA-MHB response-shift coefficient for EVO-WT and EVO-MAC comparisons. These medium-interaction tests were interpreted as exploratory processed-expression interaction analyses.

For each evolutionary state, the acute vancomycin response was defined as the expression difference between vancomycin-treated and matched untreated samples. Response rewiring was then quantified by comparing the EVO acute response with the corresponding WT or MAC acute response within the same medium. These contrasts yielded EVO-WT and EVO-MAC response-shift estimates, respectively. In addition, a basal evolved-state contrast was calculated by comparing untreated EVO and untreated MAC samples. For genome-wide summary plots, features passing the exploratory processed-expression FDR threshold of 0.05 were counted and separated according to the sign of the limma logFC estimate. This threshold was used for within-analysis ranking and count summaries, not as a calibrated raw-count RNA-seq differential-expression criterion. No additional absolute logFC threshold was applied.

### 2.4 RefSeq locus-tag harmonization and candidate evidence ranking

Candidate definition, annotation harmonization and downstream evidence ranking were treated as separate steps. The present analysis did not perform a *de novo* genome-wide reduction from all 2,888 processed GSE149213 genes to nine candidates. Instead, the nine-candidate set was treated as a predefined, locus-tag-trackable panel carried forward from prior GSE149213 response-rewiring candidate records. A record was eligible for RefSeq audit only if it could be linked to a USA300HOU RefSeq locus tag in the processed discovery matrix. GSE254530 and RNase III-CLASH evidence were not used to define this panel; they were applied only after candidate definition as independent evidence layers. Candidate identities were then harmonized using USA300HOU RefSeq locus tags as stable genomic anchors, separating locus-level tracking from annotation-source-specific display names. For each retained locus, the legacy display label was audited against the corresponding USA300HOU RefSeq locus-tag annotation and updated to the current RefSeq gene symbol or product description when appropriate.

The stepwise workflow was recorded as a reproducibility table. The processed GSE149213 matrix contained 2,888 genes available for genome-wide exploratory response modeling, and all 2,888 genes were evaluated in the processed-expression contrast analysis. The predefined candidate panel contained nine locus-tag-trackable records; all nine entered the RefSeq audit and were retained as unique RefSeq-corrected candidate loci. Downstream GSE254530 and RNase III-CLASH analyses therefore did not constitute candidate-selection filters, but post-definition evidence layers for conservative cross-dataset support and candidate-associated RNA-interaction support. Candidate-level response summaries were calculated from GSE149213 only: the EVO-WT response shift was calculated within each medium as the EVO acute response minus the WT acute response, and the maximum absolute EVO-WT response shift was defined as the larger absolute shift across CA-MHB and RPMI + 10% LB. A candidate was considered response-rewired in a given medium when the absolute EVO-WT response shift was at least 0.5. This threshold was used as a descriptive effect-size benchmark on the log2(TPM + 1)-derived response scale to avoid classifying very small response differences as rewiring; it was not used to define the nine-candidate panel, assign statistical significance or replace limma-derived genome-wide and module-level evidence. The full candidate workflow, retained counts and post-selection evidence classifications are provided in Supplementary Table 7.

### 2.5 Conservative cross-dataset assessment using GSE254530

GSE254530 was used as an independent clean vancomycin-response RNA-seq reference for conservative cross-dataset assessment of the RefSeq-corrected prioritized candidates. This assessment was performed at the level of RefSeq-corrected candidate identity rather than the legacy display label in the processed discovery matrix. Candidates were first assessed for conservative mappability to the clean GSE254530 annotation. Only candidates with a unique conservative clean-feature mapping were then evaluated in the VAN30 DESeq2 differential-expression table. Candidates without such a unique conservative mapping were excluded from differential-expression interpretation in GSE254530 rather than counted as non-responsive candidates.

A candidate was considered to have strict cross-dataset differential-expression support only if it was conservatively mapped and differentially expressed in the clean VAN30 comparison after false-discovery-rate correction. Candidates that were uniquely mapped but did not pass the differential-expression threshold were classified as mapped non-DE. Candidates without a unique conservative mapping to the clean GSE254530 annotation were not treated as having either positive or negative cross-dataset differential-expression support, but were instead classified as not conservatively mapped. This category indicates a mapping limitation under the clean GSE254530 annotation and conservative remapping rules, not evidence that the corresponding candidate lacks a vancomycin response. This framework was used to avoid overinterpreting GSE254530 as a universal support source for all prioritized candidates.

### 2.6 Candidate response trajectory analysis

Candidate-level vancomycin response trajectories were calculated for the nine RefSeq-corrected prioritized candidates in GSE149213. For each candidate, culture medium and evolutionary state, acute response was defined as vancomycin-treated minus untreated log2(TPM + 1) expression. To address the non-equivalent replicate structure of the dataset, response values were first calculated at the paired replicate or evolved-lineage/population level where matched untreated and vancomycin-treated samples were available, and were then summarized as group-level means for WT, MAC and the analytically pooled EVO state in CA-MHB and RPMI + 10% LB.

Trajectory plots were used to visualize how candidate-level acute responses changed across WT, MAC and the analytically pooled EVO state. Individual paired replicate-level responses for WT and MAC and paired evolved-lineage/population responses for EVO were overlaid on the group mean trajectories, with standard errors shown for the group summaries. Plotted values therefore represent observed response summaries rather than limma model coefficients. For each candidate and medium, response rewiring was summarized as the EVO-WT response shift, calculated as the EVO acute response minus the WT acute response. The maximum absolute EVO-WT response shift across the two media was used as the candidate-level response-rewiring summary metric. To assess whether pooled EVO-WT response shifts were dominated by any single evolved lineage, we performed a leave-one-lineage-out sensitivity analysis. In each iteration, paired responses from one EVO lineage were removed, the EVO mean response was recalculated from the remaining lineages, and the EVO-WT response shift was recomputed. Sign retention and retention of the descriptive |EVO-WT response shift| > = 0.5 benchmark were recorded for each candidate-medium combination.

### 2.7 RNase III-CLASH integration and retained interaction support

RNA-interaction evidence was integrated using the published *S. aureus* RNase III-CLASH interactome dataset from Mediati et al. (GSE158830). The analysis focused on candidate-associated RNA interactions rather than reconstruction of the full CLASH network. RefSeq-corrected prioritized candidates were matched to candidate-partner RNA pairs in the CLASH-derived interaction table, and only high-confidence interactions were retained for downstream summaries.

Retained CLASH edges were defined as candidate-partner RNA pairs with high-confidence CLASH evidence after removal of non-interpretable partner entries. High-confidence evidence was defined by CLASH FDR < 0.05. Partner entries annotated as missing, unknown, none or otherwise non-informative were excluded before defining interpretable retained edges. Retained edges were further classified by evidence tier: Tier 1 edges had FDR < 0.05 with replicated CLASH support, whereas Tier 2 edges had FDR < 0.05 without replicated support.

For each retained candidate-partner RNA edge, hybrid support was summarized as the edge-level hybrid count. Candidate-level retained CLASH support was then calculated as the sum of hybrid counts across retained interpretable high-confidence edges for that candidate. The number of Tier 1 retained edges was also recorded for each candidate. Candidate-level ranking and edge-level candidate-associated CLASH support plots were generated from these retained CLASH support summaries; these summaries were not interpreted as full-network centrality measures. To add partner-level transcriptional context, retained interpretable CLASH partner RNAs were additionally cross-referenced with the GSE149213 processed-expression response-rewiring contrast table. Partner RNAs were mapped to GSE149213 features conservatively by shared RS locus-tag suffix when the corresponding USA300HOU_RS feature was present in the processed matrix. For mapped partners, EVO-WT and EVO-MAC response-shift logFC, nominal *p* value and FDR were extracted separately for CA-MHB and RPMI + 10% LB. This partner-level annotation was used only as a secondary description of retained CLASH edges and was not used to define candidate selection, infer regulatory direction or estimate full-network centrality (Supplementary Tables 17–20).

### 2.8 Functional module scoring and module-level response-rewiring analysis

To assess whether candidate-level response rewiring extended to broader functional programs, genes were assigned to eight manually curated functional modules based on RefSeq-linked gene symbols, product annotations and broad functional categories. The modules represented membrane/transport, nucleotide/RNA metabolism, proteolysis/stress/virulence, translation/ribosome, cell wall/envelope, DNA replication/repair, central metabolism/respiration and redox/stress programs. The complete recovered module membership table, including locus tags, gene symbols, product annotations and assigned module categories, is provided in Supplementary Table 6. These modules were used as interpretable annotation-based summaries rather than as GO, KEGG or COG-derived gene sets. As a complementary pathway-level check, Kyoto Encyclopedia of Genes and Genomes (KEGG) pathway overrepresentation analysis was performed for features passing the exploratory processed-expression FDR threshold of 0.05 in the EVO-WT response-shift contrast within each medium (Kanehisa et al., 2023). USA300HOU_RS locus tags were mapped to KEGG orthology/pathway identifiers through KEGG REST outputs, and overrepresentation was tested using a hypergeometric framework against the KEGG-mapped GSE149213 background. KEGG results were used only as an orthogonal pathway-level check on the manually curated modules, not as the primary basis for module construction or candidate selection.

Module scores were calculated from the GSE149213 log2(TPM + 1) expression matrix. For each gene, expression values were standardized across samples, and module scores were then computed as the mean standardized expression of all genes assigned to a given module. This produced a module-score matrix with modules as rows and samples as columns. For visualization of module trajectories, module response was summarized for each medium and evolutionary state as the mean module score in vancomycin-treated samples minus the mean module score in matched untreated samples.

Module-level response-rewiring statistics were estimated using limma on the module-score matrix. As in the gene-level analysis, CA-MHB and RPMI + 10% LB samples were analysed separately with a no-intercept design based on evolutionary state and treatment. EVO-WT and EVO-MAC module response shifts were estimated by comparing the EVO acute module response with the corresponding WT or MAC acute module response. *p* values were obtained from moderated limma statistics, and FDR values were calculated using Benjamini-Hochberg correction. In the module heatmap, an asterisk denotes FDR < 0.05, whereas a dagger denotes nominal *p* < 0.05 with FDR ≥ 0.05.

### 2.9 Visualization and software

All statistical analyses and visualizations were performed in R. Differential-expression and response-rewiring analyses were performed with limma. Heatmaps, bar plots, trajectory plots and multi-panel figures were generated using R plotting packages, including ggplot2, ComplexHeatmap and patchwork or cowplot where appropriate. Figure panels were assembled and exported as publication-resolution raster images and vector files when available.

For all statistical summaries, FDR refers to Benjamini-Hochberg-adjusted *p-*values unless otherwise stated. Genome-wide response and response-rewiring feature counts were reported as the number of features passing the exploratory processed-expression FDR threshold of 0.05, without an additional absolute logFC cutoff. Candidate response trajectories and module response trajectories were plotted from group-level response summaries, whereas genome-wide and module-level statistical evidence was derived from limma contrast estimates. Because the analysis used processed expression values reported as TPM rather than raw read counts, genome-wide FDR-ranked contrasts are interpreted as exploratory processed-expression reanalysis rather than as definitive raw-count differential-expression results. Accordingly, genome-wide FDR thresholds in GSE149213 were used as processed-expression ranking and count-summary criteria, not as calibrated raw-count RNA-seq differential-expression calls. To improve reproducibility, analysis scripts, an output manifest and R session information are provided as Supplementary code, and package versions are summarized in Supplementary Table 12. Complete candidate definitions, RefSeq audit fields, candidate workflow criteria and module membership are provided in Supplementary Tables 1, 6, 7.

## 3 Results

### 3.1 Medium-stratified transcriptomic structure frames vancomycin response rewiring in GSE149213

To establish a discovery framework for vancomycin response rewiring, we re-analysed GSE149213, which profiles wild-type (WT), media-adapted control (MAC) and analytically pooled vancomycin-evolved (EVO) lineages/populations across cation-adjusted Mueller-Hinton broth (CA-MHB) and Roswell Park Memorial Institute medium supplemented with 10% lysogeny broth (RPMI + 10% LB), with untreated and acute vancomycin-treated conditions. The EVO state comprised multiple evolved strain/lineage labels pooled analytically rather than replicate measurements of one evolved clone. Sample-level Pearson correlation analysis revealed a structured transcriptomic landscape, with clustering patterns aligned with culture medium, evolutionary state and treatment annotations (Figure 1A and Supplementary Table 2). This organization supported a medium-stratified analysis strategy rather than pooled modeling across culture conditions.

**Figure 1:**
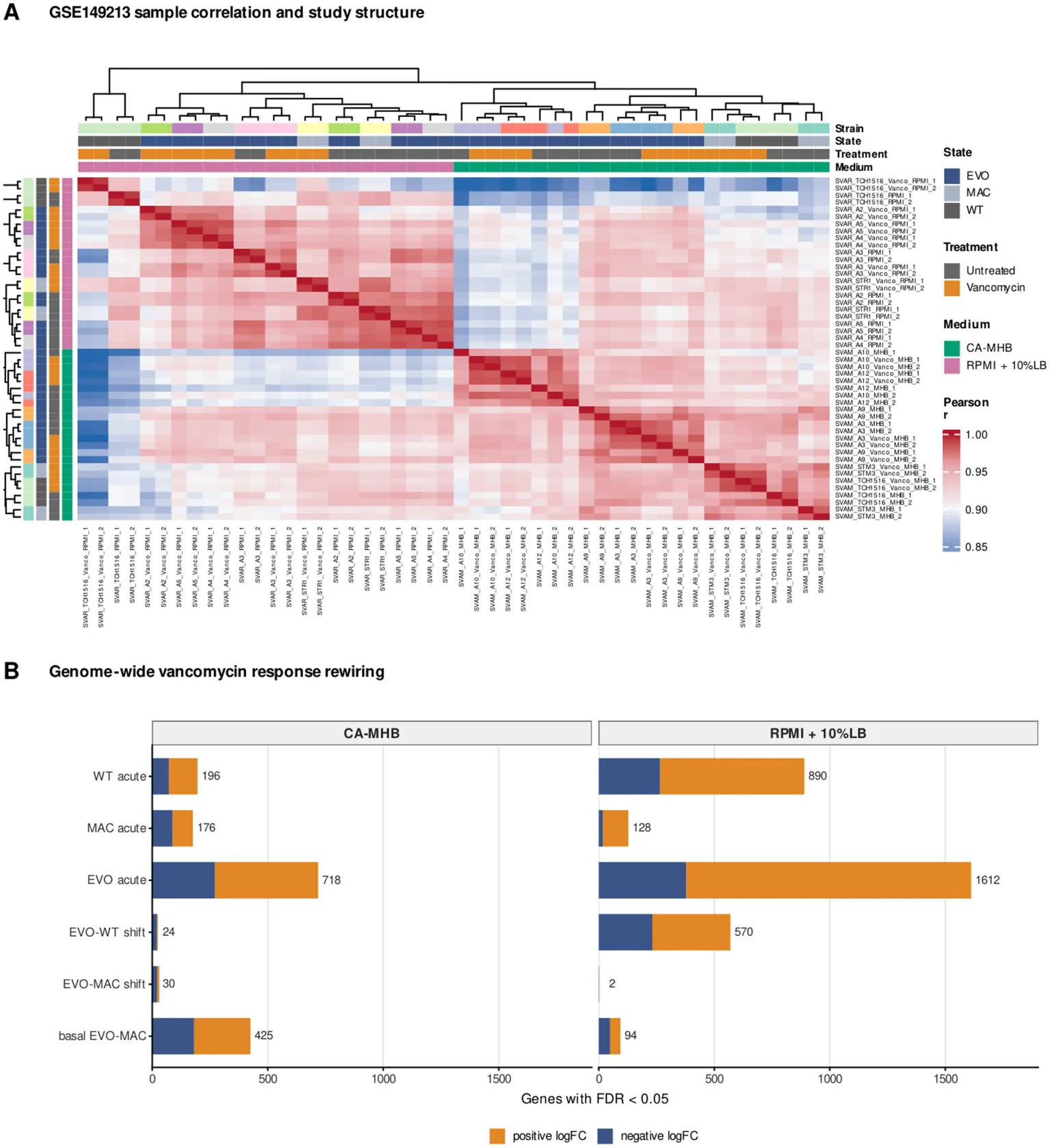
GSE149213 sample structure and genome-wide vancomycin response rewiring. **(A)** Sample-level Pearson correlation heatmap of 48 GSE149213 RNA-seq samples, annotated by strain, evolutionary state, treatment, and medium. Rows and columns were ordered by hierarchical clustering. For EVO, each medium/treatment group comprised four evolved strain/lineage labels with two samples each; EVO was therefore treated as an analytically pooled evolved-lineage/population group rather than as replicate measurements of a single evolved clone. **(B)** Genome-wide counts of features passing the exploratory processed-expression FDR threshold of 0.05 in Limma contrasts for acute vancomycin responses or response-rewiring contrasts, stratified by medium and logFC direction. These counts were derived from log2(TPM + 1) processed-expression values rather than raw-count RNA-seq differential-expression modeling. Acute response was defined as vancomycin-treated minus untreated expression. WT, wild-type; MAC, media-adapted control.

We then quantified genome-wide acute vancomycin responses and response-rewiring contrasts using exploratory FDR-adjusted processed-expression contrast counts. Acute response was defined as vancomycin-treated minus untreated expression within each evolutionary state, and counts were summarized by logFC direction. In CA-MHB, the number of features passing the exploratory processed-expression FDR threshold increased from 196 in WT (124 positive and 72 negative logFC features) and 176 in MAC (88 positive and 88 negative) to 718 in EVO (447 positive and 271 negative). A stronger expansion was observed in RPMI + 10% LB, where WT, MAC and EVO showed 890 features (625 positive and 265 negative), 128 features (110 positive and 18 negative) and 1,612 features (1,234 positive and 378 negative), respectively (Figure 1B and Supplementary Table 3). Thus, within this processed-expression framework, the analytically pooled evolved lineages/populations retained broad acute vancomycin responsiveness, with the largest genome-wide response burden detected under RPMI + 10% LB.

Response rewiring relative to ancestral or control states was strongest in the RPMI + 10% LB EVO-WT contrast rather than evenly distributed across media or control comparisons. The EVO-WT response shift involved only 24 features passing the exploratory processed-expression FDR threshold in CA-MHB (4 positive and 20 negative logFC features) but 570 in RPMI + 10% LB (338 positive and 232 negative). By contrast, EVO-MAC response shifts were limited in both media, with 30 features in CA-MHB (9 positive and 21 negative) and 2 features in RPMI + 10% LB (1 positive and 1 negative). Basal EVO-MAC contrasts involved 425 features in CA-MHB (244 positive and 181 negative) and 94 features in RPMI + 10% LB (45 positive and 49 negative). We therefore performed a direct medium-interaction test comparing response-shift coefficients between RPMI + 10% LB and CA-MHB. This interaction analysis identified 89 features meeting the exploratory processed-expression FDR threshold for the EVO-WT medium interaction, compared with 15 features for the EVO-MAC medium interaction (Supplementary Table 8). These results support the interpretation that, within the processed-expression reanalysis, the strongest medium-associated response-rewiring signal in GSE149213 was the RPMI + 10% LB EVO-WT shift, whereas response shifts relative to MAC were comparatively restricted. Because EVO comprised pooled evolved lineages/populations, medium-associated effects cannot be fully separated from lineage composition in this processed-expression reanalysis.

### 3.2 RefSeq locus-tag harmonization defines nine prioritized candidates for cross-dataset integration

To enable consistent integration across transcriptomic response modeling, conservative cross-dataset assessment and RNA-interaction evidence, we first fixed a predefined nine-locus candidate panel from locus-tag-trackable GSE149213 response-rewiring candidate records and then harmonized candidate identities using USA300HOU RefSeq locus tags as stable genomic anchors. This step separated locus-level candidate tracking from legacy display-name variability in the processed discovery matrix and allowed candidate response trajectories, GSE254530 mapping/DE status and CLASH support to be evaluated on the same RefSeq-corrected identifiers (Table 1 and Supplementary Tables 1, 7). GSE254530 and RNase III-CLASH evidence were not used to define or select the nine-candidate panel, but were applied after candidate definition as evidence-ranking layers.

**Table 1:** RefSeq-corrected prioritized vancomycin-response candidates.

Evidence tier	RefSeq-corrected candidate	GSE149213 response rewiring	Clean GSE254530 support	Retained RNase III-CLASH support
Cross-dataset DE support	pyrR USA300HOU_RS06020	2.20; 2/2 media	DE; log2FC -1.68; FDR 1.9e-9	0 hybrids; 0 Tier 1 edges
Candidate-associated CLASH hotspot	carbamoyl-P synthase USA300HOU_RS06040	4.60; 2/2 media	Not conservatively mapped	56 hybrids; 2 Tier 1 edges
Candidate-associated CLASH hotspot	dihydroorotase USA300HOU_RS06035	5.20; 2/2 media	Not conservatively mapped	46 hybrids; 0 Tier 1 edges
Candidate-associated CLASH hotspot	glnA USA300HOU_RS06590	1.35; 1/2 media	Mapped non-DE; FDR 0.128	29 hybrids; 1 Tier 1 edge
Additional CLASH-supported candidate	icd USA300HOU_RS08980	0.55; 1/2 media	Not conservatively mapped	15 hybrids; 0 Tier 1 edges
Additional CLASH-supported candidate	GDE phosphodiesterase USA300HOU_RS05595	1.53; 1/2 media	Not conservatively mapped	9 hybrids; 0 Tier 1 edges
Additional CLASH-supported candidate	ABC transporter permease USA300HOU_RS05490	1.82; 1/2 media	Not conservatively mapped	1 hybrid; 0 Tier 1 edges
Response-rewiring only	PLP aminotransferase USA300HOU_RS09140	2.22; 2/2 media	Not conservatively mapped	0 hybrids; 0 Tier 1 edges
Response-rewiring only	DUF4242 protein USA300HOU_RS00905	1.93; 2/2 media	Not conservatively mapped	0 hybrids; 0 Tier 1 edges

GSE149213 response rewiring is reported as max |EVO-WT response shift| across CA-MHB and RPMI + 10% LB, followed by the number of media meeting the descriptive response-shift criterion (|EVO-WT response shift| > = 0.5). This criterion summarizes candidate-level effect size and should not be interpreted as a statistical significance threshold. Clean GSE254530 was used as a conservative cross-dataset support dataset, not as a universal confirmation source; candidates without a unique conservative clean-feature mapping were classified as not conservatively mapped rather than as GSE254530-negative. Retained CLASH support is summarized as summed hybrid count and number of Tier 1 retained edges after filtering for interpretable high-confidence candidate-partner edges. EVO denotes analytically pooled evolved lineages/populations.

DE, differentially expressed; FDR, false discovery rate; CLASH, crosslinking, ligation and sequencing of hybrids; EVO, vancomycin-evolved state.

The RefSeq audit retained nine unique candidate loci: pyrR at USA300HOU_RS06020, carbamoyl-P synthase at USA300HOU_RS06040, dihydroorotase at USA300HOU_RS06035, glnA at USA300HOU_RS06590, icd at USA300HOU_RS08980, GDE phosphodiesterase at USA300HOU_RS05595, ABC transporter permease at USA300HOU_RS05490, PLP aminotransferase at USA300HOU_RS09140 and a DUF4242 protein at USA300HOU_RS00905. By anchoring candidates to RefSeq locus tags, subsequent analyses were performed on a consistent candidate set rather than on annotation-source-specific display names. The stepwise retained counts are summarized in Supplementary Table 7.

We then used clean GSE254530 as an independent vancomycin-response RNA-seq reference for conservative cross-dataset assessment. Under this conservative remapping framework, only pyrR showed strict cross-dataset differential-expression support in the clean GSE254530 VAN30 DESeq2 table (log2FC = −1.68, FDR = 1.89 × 10^-9). glnA was uniquely mapped but was not differentially expressed (log2FC = −0.247, FDR = 0.128), whereas the remaining seven candidates were not conservatively mapped to the clean GSE254530 annotation. These seven candidates were excluded from GSE254530 differential-expression interpretation because no unique conservative clean-feature match was retained under the RefSeq-corrected mapping framework; this should not be interpreted as evidence against their response behavior in GSE149213. Thus, clean GSE254530 provided strict cross-dataset DE support for one candidate only, while the full nine-candidate set remained a RefSeq-corrected prioritization framework for response and interaction analyses.

### 3.3 RefSeq-corrected candidates show divergent vancomycin-response trajectories across adaptation states

We next examined whether the RefSeq-corrected prioritized candidates showed altered acute vancomycin-response patterns across adaptation states. For each candidate, response was defined as vancomycin-treated minus untreated expression and was compared across WT, MAC and the analytically pooled EVO lineages/populations in CA-MHB and RPMI + 10% LB. To make the EVO structure explicit, Figure 2 shows individual paired evolved-lineage/population responses together with group mean trajectories. This analysis revealed heterogeneous response trajectories rather than a uniform evolved-state pattern, indicating that the prioritized candidates captured multiple modes of response rewiring (Figure 2 and Table 1).

**Figure 2:**
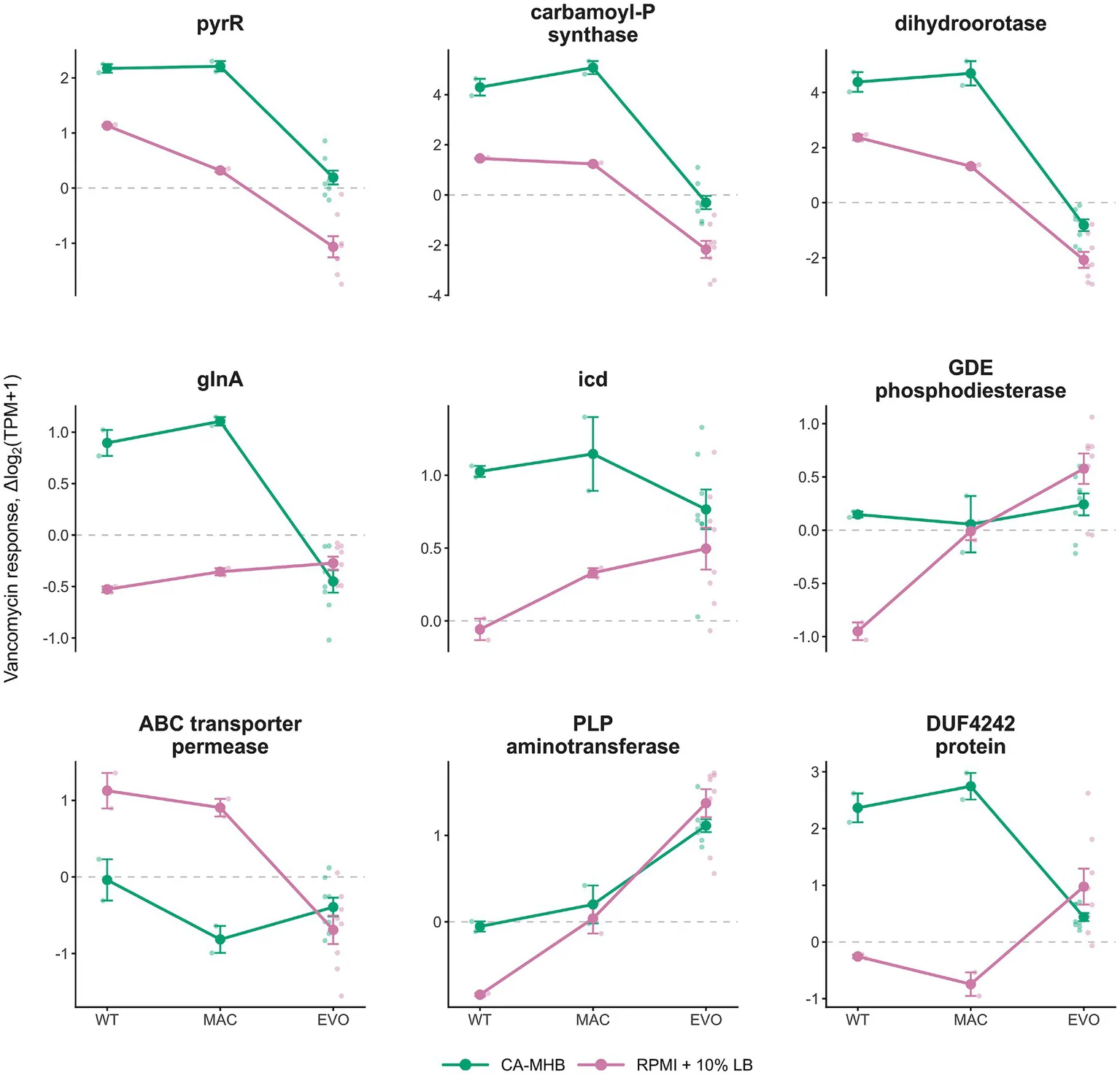
RefSeq-corrected candidate response trajectories across vancomycin adaptation states. Vancomycin response trajectories are shown for nine RefSeq-corrected prioritized candidates across WT, MAC and the analytically pooled EVO state. Response was defined as vancomycin-treated minus untreated expression and is shown as Δlog2(TPM + 1). Thin points represent paired replicate-level responses for WT and MAC or paired evolved-lineage/population responses for EVO; bold points and lines show group means with standard errors. Green indicates cation-adjusted Mueller-Hinton broth (CA-MHB) and pink indicates Roswell Park Memorial Institute medium supplemented with 10% lysogeny broth (RPMI + 10% LB). Facets use independent *y*-axis ranges. EVO denotes analytically pooled evolved lineages/populations rather than replicate measurements of a single evolved clone. Lineage-level response summaries and leave-one-lineage-out sensitivity results are provided in Supplementary Tables 21, 22.

Several candidates showed marked attenuation or reversal of vancomycin response in the analytically pooled EVO group. pyrR, carbamoyl-P synthase, dihydroorotase and glnA showed strong positive responses in one or both ancestral/control states but weaker or negative mean responses in EVO, consistent with evolved-state dampening of acute response at the pooled-population level. In contrast, PLP aminotransferase and the DUF4242 protein showed increased EVO-associated responses, whereas GDE phosphodiesterase and ABC transporter permease displayed medium-associated or directionally divergent patterns. Overall, five of the nine RefSeq-corrected candidates showed response rewiring in both media. The overlaid EVO lineage/population points showed within-group dispersion, so these patterns should be interpreted as evolved-population trends rather than uniform responses shared by every evolved lineage. Leave-one-lineage-out sensitivity analysis nevertheless supported the stability of the candidate-level signal: all 18 candidate-medium EVO-WT response shifts retained their direction after exclusion of any single EVO lineage, and 13 of 14 candidate-medium shifts that met the descriptive |EVO-WT response shift| > = 0.5 benchmark in the full analysis retained this benchmark across all lineage-removal iterations. The only threshold-sensitive case was the RPMI + 10% LB icd shift, which was close to the 0.5 benchmark in the full analysis. The five candidates satisfying the 2/2 medium response-rewiring criterion in the pooled analysis retained this benchmark after leave-one-lineage-out analysis in both media. Together, these results support candidate-level remodeling of vancomycin response programs while preserving the interpretation that the EVO signal represents analytically pooled evolved-lineage/population trends rather than clone-level convergence (Figure 2, Table 1 and Supplementary Tables 21, 22).

Thus, candidate-level trajectories separated candidates with strict cross-dataset differential-expression support, such as pyrR, from candidates whose post-selection evidence profile was driven mainly by GSE149213 response dynamics and, where present, retained RNA-interaction evidence. This trajectory-based view provided a response-level framework for evaluating which RefSeq-corrected candidates were further supported by CLASH-derived RNA-interaction data.

### 3.4 RNase III-CLASH evidence nominates candidate-associated RNA-interaction hotspots

We next asked whether the RefSeq-corrected prioritized candidates were supported by RNA-interaction evidence. To do this, we integrated the candidate set with the published *S. aureus* RNase III-CLASH RNA–RNA interactome dataset from Mediati et al. (GSE158830), focusing on retained interpretable high-confidence CLASH edges. This filtering strategy was designed to prioritize candidate-associated RNA interactions that could be conservatively assigned to interpretable partner RNAs, rather than to reconstruct the full CLASH network.

Among the nine RefSeq-corrected candidates, six retained CLASH support after filtering. Candidate-level ranking identified three prominent candidate-associated CLASH hotspots: carbamoyl-P synthase, with 56 retained hybrids including two Tier 1 edges; dihydroorotase, with 46 retained hybrids; and glnA, with 29 retained hybrids including one Tier 1 edge (Figure 3A and Supplementary Table 4). Three additional candidates, icd, GDE phosphodiesterase and ABC transporter permease, had lower retained CLASH support, whereas pyrR, PLP aminotransferase and the DUF4242 protein did not retain interpretable CLASH edges in this filtered analysis. These rankings describe retained candidate-associated CLASH support and should not be interpreted as full-network centrality.

**Figure 3:**
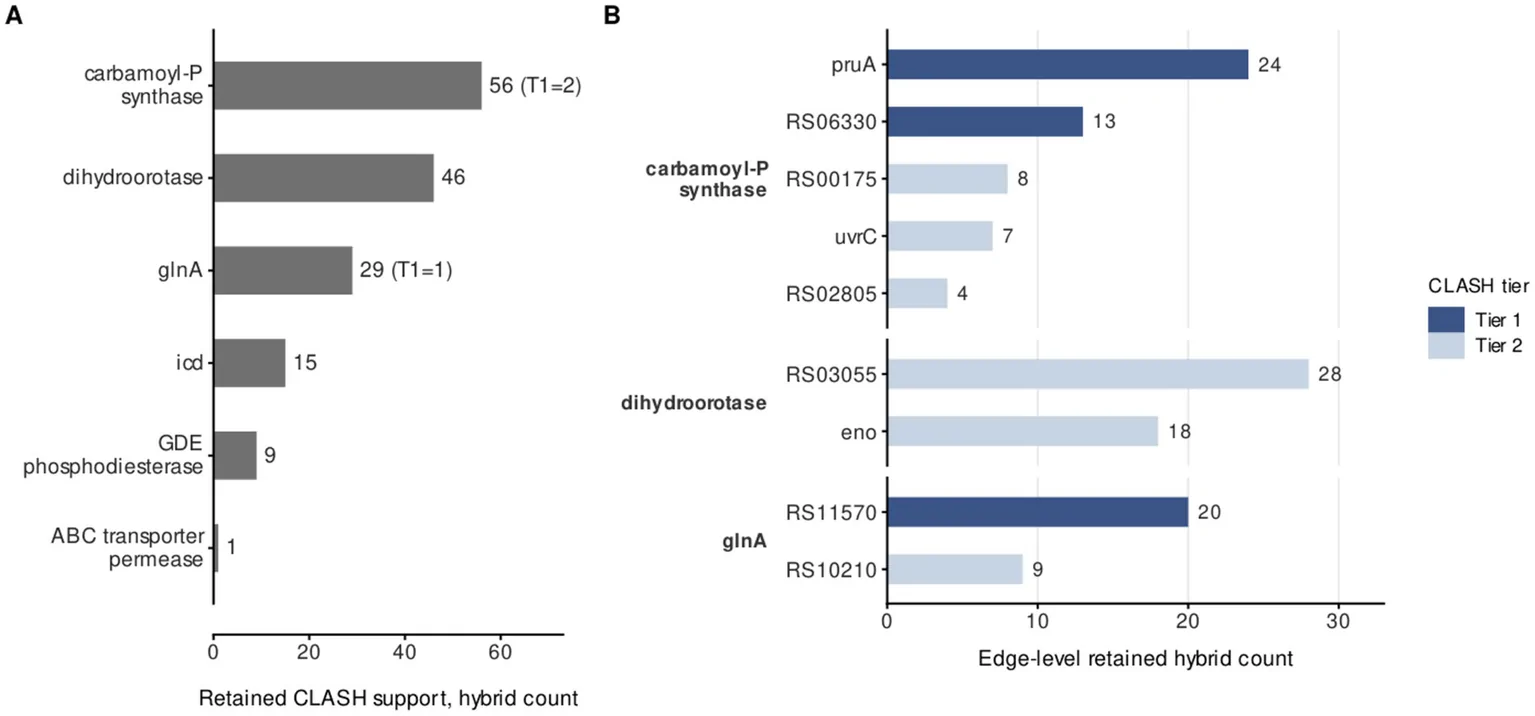
Retained CLASH support identifies candidate-associated RNA-interaction hotspots among RefSeq-corrected prioritized candidates. **(A)** Candidate-level ranking of retained CLASH-supported hybrids among prioritized candidates with non-zero retained CLASH support. Labels show total retained hybrid count and Tier 1 count where applicable. **(B)** Edge-level retained interpretable high-confidence CLASH partner RNAs for the three top-ranked candidate-associated CLASH hotspots. Bar length indicates retained hybrid count, and color indicates CLASH evidence tier. These rankings summarize retained candidate-associated CLASH support rather than full-network centrality. Locus-tagged partner RNAs were retained where no reliable gene symbol or product annotation was available; placeholder or non-interpretable partner entries were excluded.

Edge-level inspection further localized these candidate-associated CLASH signals to a limited set of partner RNAs. Carbamoyl-P synthase was linked to pruA, RS06330, RS00175, uvrC and RS02805; dihydroorotase was linked to RS03055 and eno; and glnA was linked to RS11570 and RS10210 (Figure 3B). Because several partner RNAs lacked reliable gene-symbol or product annotation in the exported results, these were retained as locus-tagged partner RNAs rather than assigned speculative product labels. Together, these data nominate carbamoyl-P synthase, dihydroorotase and glnA as RefSeq-corrected candidates with both response-rewiring behavior and retained RNA-interaction support.

To determine whether retained CLASH partners also showed transcriptional response-shift evidence in GSE149213, we annotated the retained interpretable high-confidence candidate-partner edges against the processed-expression contrast results. Of 14 retained interpretable high-confidence CLASH edges, 12 could be mapped back to GSE149213 features by shared RS locus-tag suffix. Partner-level response-shift support was limited rather than global: one mapped edge reached FDR < 0.05 in the CA-MHB EVO-WT contrast, one mapped edge reached FDR < 0.05 in the CA-MHB EVO-MAC contrast, two mapped edges reached FDR < 0.05 in the RPMI + 10% LB EVO-WT contrast and no mapped partner edge reached FDR < 0.05 in the RPMI + 10% LB EVO-MAC contrast (Supplementary Tables 17–20). The strongest partner-level signal involved uvrC, a carbamoyl-P synthase-associated Tier 2 partner, which showed significant response shifts in CA-MHB and RPMI + 10% LB EVO-WT contrasts; RS00175 also showed an RPMI + 10% LB EVO-WT response shift. A Fisher exact context test did not show significant enrichment of mapped CLASH partners among response-shift genes after FDR correction. Thus, the CLASH layer provides prior physical RNA-interaction evidence with limited partner-level transcriptional support, rather than broad enrichment of rewired partner RNAs.

### 3.5 Module-level analysis localizes evolved response rewiring to RPMI-enriched stress and envelope programs

We next asked whether the candidate-level response patterns were reflected at the functional-module level. For each annotation-curated module, we calculated a vancomycin response score as the difference between vancomycin-treated and untreated module scores, and then tested evolved-state response shifts relative to WT and MAC populations. Positive shifts indicate increased evolved-state vancomycin response, whereas negative shifts indicate reduced evolved-state response (Figure 4A and Supplementary Table 5). Complete module membership and recovered gene counts are provided in Supplementary Table 6, so these results should be interpreted as module-level summaries of predefined functional categories rather than as *de novo* pathway-enrichment results. As an orthogonal pathway-level check, we tested KEGG pathway overrepresentation among features passing the exploratory processed-expression FDR threshold in the EVO-WT response-shift contrast within each medium.

**Figure 4:**
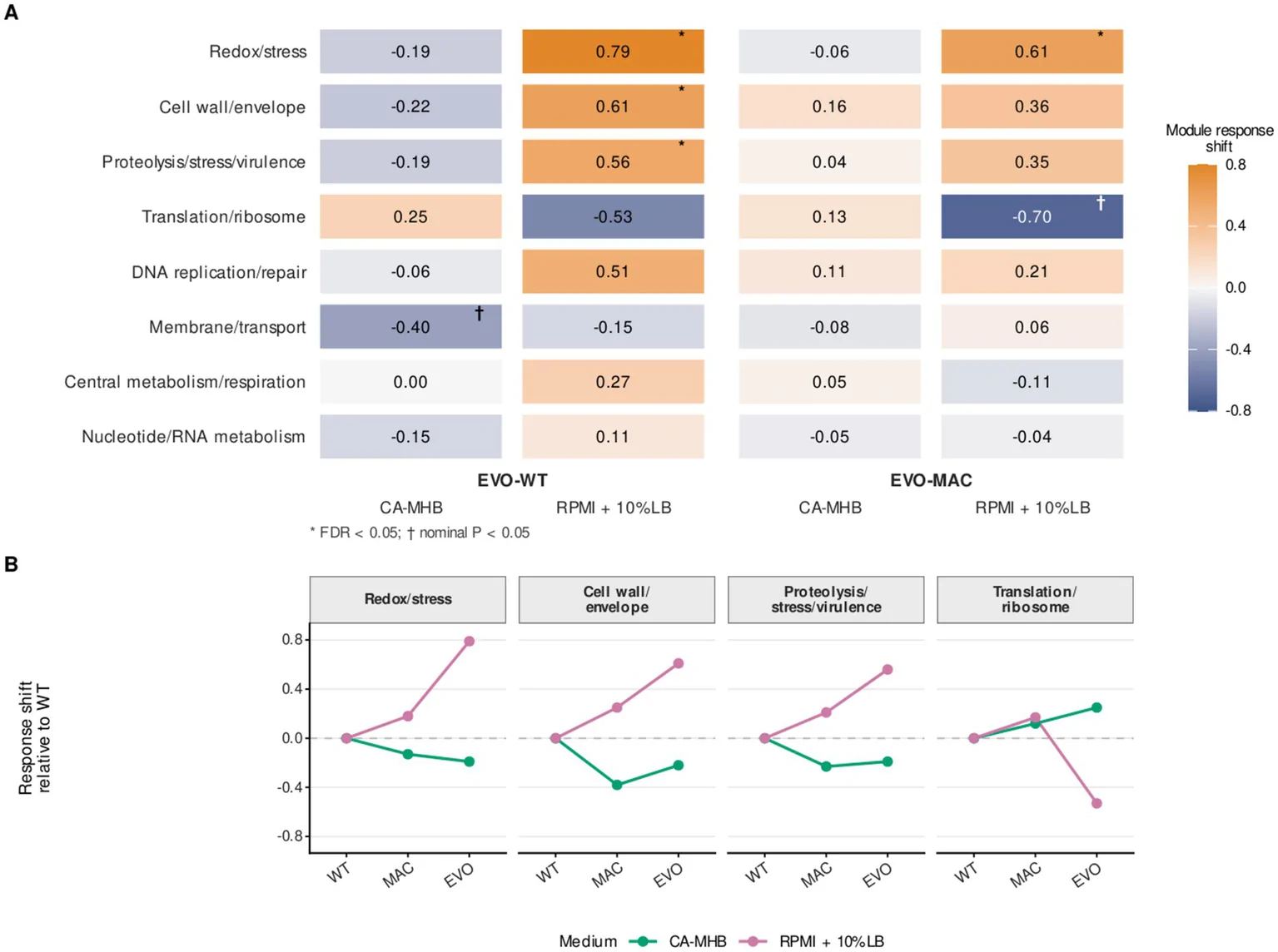
Module-level vancomycin response rewiring is strongest in RPMI-associated EVO-WT contrasts. **(A)** Heatmap of module-level response shifts in GSE149213, calculated from vancomycin-treated minus untreated module response scores. Values indicate EVO response shifts relative to WT or MAC; the EVO state represents analytically pooled evolved lineages/populations. Positive values indicate increased evolved-state vancomycin response, whereas negative values indicate reduced response. *FDR < 0.05; ^†^nominal *p* < 0.05. **(B)** Contrast-derived response-shift trajectories relative to WT for representative modules showing RPMI-enriched rewiring patterns. Green indicates cation-adjusted Mueller-Hinton broth (CA-MHB) and pink indicates Roswell Park Memorial Institute medium supplemented with 10% lysogeny broth (RPMI + 10% LB). Because EVO comprised pooled evolved lineages/populations, medium-associated effects should be interpreted as evolved-population signals rather than clone-level effects.

Module-level rewiring was strongest in RPMI + 10% LB. In the EVO-WT comparison, redox/stress showed the largest positive shift (logFC = 0.791, FDR = 0.00164), followed by cell wall/envelope (logFC = 0.611, FDR = 0.0318) and proteolysis/stress/virulence (logFC = 0.559, FDR = 0.0318). These modules therefore showed FDR-level evidence for increased evolved-state vancomycin response under RPMI + 10% LB. By contrast, CA-MHB showed weaker module-level evidence, with membrane/transport showing only nominal evidence for a reduced EVO-WT response shift (logFC = −0.403, *p* = 0.0158, FDR = 0.126).

The EVO-MAC contrast was more limited but still identified selected module-level differences. Redox/stress remained significantly increased in RPMI + 10% LB (logFC = 0.611, FDR = 0.0209), whereas translation/ribosome showed a negative nominal shift (logFC = −0.698, *p* = 0.0148, FDR = 0.0594). Representative trajectories illustrated these patterns, with RPMI + 10% LB showing increased evolved-state responses in redox/stress, cell wall/envelope and proteolysis/stress/virulence modules, but a reduced EVO response in the translation/ribosome module (Figure 4B). Together, these results extend the candidate-level analysis to a module-level framework and indicate that evolved vancomycin response rewiring is concentrated in RPMI + 10% LB, especially in EVO-WT contrasts, rather than uniformly distributed across all functional modules.

## 4 Discussion

This study reframes vancomycin adaptation in *S. aureus* as medium-associated remodeling of inducible response programs rather than as a simple catalogue of vancomycin-responsive genes. By separating acute vancomycin response from evolved-state response shifts, and by anchoring candidate interpretation to RefSeq locus tags, we integrated transcriptomic, conservative cross-dataset and RNase III-CLASH evidence within a single framework. The central observation is that analytically pooled evolved lineages/populations do not merely amplify or suppress a uniform drug-response program; instead, they redistribute vancomycin response across media, candidate loci and functional modules. This places the prioritized candidates in a clearer interpretive hierarchy: they are not presented as experimentally proven resistance determinants, but as annotation-corrected, evidence-ranked hypotheses for RNA-associated vancomycin response rewiring.

The medium-associated structure of the response-shift signal is particularly important, but it should be interpreted primarily through the EVO-WT contrast. In GSE149213, the strongest genome-wide response-rewiring burden occurred for the EVO-WT shift in RPMI + 10% LB, whereas EVO-MAC gene-level shifts were comparatively small. A direct medium-interaction analysis supported this interpretation, identifying more FDR-level medium-dependent response-shift genes for EVO-WT than for EVO-MAC. This pattern is consistent with recent evidence that host-derived or medium-defined environments can reshape *S. aureus* metabolism, antibiotic tolerance and susceptibility profiles, indicating that antimicrobial response phenotypes are not fixed properties of a strain measured under a single standard condition (Machado et al., 2021; Cheng et al., 2024; Freiberg et al., 2024). The module-level results further refine this interpretation by localizing the strongest RPMI-associated EVO-WT shifts to redox/stress, cell-wall/envelope and proteolysis/stress/virulence programs, with translation/ribosome showing a distinct negative EVO-MAC trend. Because the EVO group comprised pooled evolved lineages/populations, these medium-associated patterns should be interpreted as evolved-population signals and cannot be fully separated from lineage composition in this processed-expression reanalysis.

The RefSeq-corrected audit was also important for controlling interpretive drift across datasets. In bacterial transcriptomic analyses, gene symbols and product names can change as genome annotations are updated, whereas locus tags provide a more stable anchor for mapping features across reference builds and derived result tables (Goldfarb et al., 2025; Haft et al., 2024; Chorlton, 2024). In this study, this distinction materially affected candidate interpretation. The candidate set was therefore not treated as a simple list of legacy gene names; instead, each candidate was re-anchored to USA300HOU RefSeq locus tags and then reinterpreted using the corresponding current gene symbol or product annotation. This conservative approach narrowed cross-dataset support rather than inflating it: only pyrR carried strict differential-expression support in the clean GSE254530 VAN30 table, glnA was uniquely mapped but not differentially expressed, and the remaining seven candidates lacked unique conservative clean-feature mappings. Although this limits the strength and breadth of cross-dataset evidence, it also prevents overstatement. The resulting candidate hierarchy is therefore more defensible: pyrR carries the strongest cross-dataset differential-expression support, glnA is mapped but not supported as differentially expressed in GSE254530, and the remaining candidates are supported primarily by GSE149213 response rewiring and, where present, retained CLASH evidence.

The candidate-level trajectories further suggest that evolved vancomycin responses converge on metabolic state rather than on a single canonical resistance pathway. Several prioritized candidates map to nucleotide and nitrogen-linked processes, including pyrR, carbamoyl-P synthase, dihydroorotase and glnA, while others point to central metabolism, transport or poorly characterized protein functions. This organization is consistent with studies showing that *S. aureus* vancomycin susceptibility and antibiotic tolerance can be modulated by metabolic state, nutrient limitation and nucleotide biosynthetic capacity (Cheng et al., 2024; DiMaggio et al., 2025; Kim et al., 2023). It is also compatible with more specific work linking *S. aureus* pyrimidine availability to nucleotide pools, cell-envelope homeostasis, oxidative-stress resistance, biofilm or virulence phenotypes and Sae-dependent signaling (DiMaggio et al., 2025; Kim et al., 2023), and with genetic evidence that the glnRA/glutamine synthetase locus can influence cell-wall-active antibiotic phenotypes through glutamine availability (Gustafson et al., 1994). Importantly, the present analysis does not establish these candidates as causal resistance determinants. Instead, it identifies loci whose acute vancomycin responses are reshaped across WT, media-adapted and vancomycin-evolved backgrounds. The strongest interpretation is therefore hypothesis-generating: pyrimidine- and nitrogen-associated candidates may mark metabolic response states that are coupled to vancomycin adaptation, and their prioritization is strengthened when response rewiring aligns with strict cross-dataset support or RNA-interaction evidence.

The RNA-interaction layer adds a complementary axis to this candidate hierarchy. Because the retained edges were derived from the published RNase III-CLASH dataset of Mediati et al., CLASH-supported candidates should be interpreted as loci with prior physical RNA-interaction evidence rather than as *de novo* network predictions or inferred regulatory effects (Mediati et al., 2022). This evidence layer is biologically relevant because small regulatory RNAs have been shown to influence antibiotic resistance and adaptation in *S. aureus*, including through regulation of resistance-associated protein regulators and efflux-linked adaptive programs (Eyraud et al., 2014; Ha et al., 2025). In our analysis, retained interpretable high-confidence CLASH support was not evenly distributed across the nine RefSeq-corrected candidates, but was concentrated in carbamoyl-P synthase, dihydroorotase and glnA, defining top-ranked candidate-associated CLASH hotspots. We further annotated retained CLASH partner RNAs against GSE149213 response-shift statistics and found partner-level transcriptional support for only a subset of mapped edges, including uvrC and RS00175 among carbamoyl-P synthase-associated partners. This result strengthens the interpretation that some candidate-associated CLASH edges coincide with response-rewired partner RNAs, but it also argues against presenting the CLASH layer as a globally enriched rewired partner network. Accordingly, these candidate-associated CLASH hotspots should be viewed as experimentally tractable hypotheses that require partner-specific reporter assays, candidate or partner-RNA perturbation, and validation under matched vancomycin and medium conditions.

At the module level, the strongest response-rewiring signals place the candidate-level findings within broader stress and metabolic programs. Rather than indicating uniform remodeling of all functional categories, the module analysis showed a more selective pattern: analytically pooled evolved lineages/populations displayed increased RPMI-associated EVO-WT responses in redox/stress, cell-wall/envelope and proteolysis/stress/virulence modules, whereas the translation/ribosome module showed a negative EVO-MAC shift. This organization is biologically plausible because vancomycin adaptation in *S. aureus* has been linked to physiological and metabolic remodeling, oxidative-stress compensation and glycopeptide-associated persistence programs (Cheng et al., 2024; Tan et al., 2019; Pinel-Marie et al., 2021; Habib et al., 2023). The translation-associated signal is particularly notable, as ribosome binding and translational attenuation have been implicated in *S. aureus* persister formation (Pinel-Marie et al., 2021). Thus, the module-level analysis supports a model in which evolved vancomycin response is organized around condition-dependent stress-state remodeling rather than around a single linear resistance pathway. The CA-MHB results provide an important boundary to this interpretation: module-level rewiring was weaker in this medium, with only nominal evidence for membrane/transport response shifting, reinforcing that the strongest module-level effects are context-dependent.

These findings provide a constrained translational frame for orthopaedic infection biology. Bone and implant-associated *S. aureus* infections are shaped by heterogeneous local environments, including biofilm-associated growth, inflammatory stress, altered nutrient availability and variable antibiotic exposure1,3. The present analysis does not model those infection environments directly and should not be interpreted as evidence explaining clinical vancomycin failure. Rather, the medium-associated response-rewiring patterns observed in public *in vitro* datasets support a testable premise: antibiotic-response states may depend on environmental context and should be evaluated in biologically relevant infection models. The prioritized RefSeq-corrected candidates, candidate-associated CLASH hotspots and module-level response shifts therefore provide focused hypotheses for future validation in implant biofilm, osteoblast- or osteocyte-associated infection and local vancomycin exposure systems.

Several limitations should temper the interpretation of these findings. This study is a secondary integration of public transcriptomic and RNA-interaction datasets, and therefore identifies transcriptional associations and candidate RNA-interaction evidence rather than causal resistance mechanisms. First, all genome-wide and module-level statistical summaries were derived from the available processed TPM expression matrix transformed as log2(TPM + 1), because raw read-count matrices were not available among the retrieved GEO processed files. The FDR-adjusted limma contrasts, including the medium-interaction tests, should therefore be interpreted as exploratory processed-expression evidence rather than calibrated raw-count RNA-seq differential-expression results. Second, the GSE149213 design is informative but unbalanced: WT and MAC included two biological replicates per medium/treatment condition, whereas the EVO group comprised multiple evolved lineages/populations that were analyzed as an analytically pooled group. Consequently, EVO-associated response shifts should be viewed as pooled evolved-population signals; although the leave-one-lineage-out analysis supported the direction stability of candidate-level EVO-WT response shifts, it does not demonstrate clone-level convergent evolution and may still reflect a mixture of shared adaptive responses, lineage-specific expression states and lineage composition. Conservative GSE254530 remapping improved specificity but reduced sensitivity, leaving pyrR as the only candidate with strict cross-dataset differential-expression support and leaving seven candidates outside interpretable GSE254530 DE assessment because no unique conservative clean-feature mapping was retained. RNase III-CLASH support indicates physical or proximity-based RNA-interaction evidence, not regulatory direction, interaction stoichiometry or phenotypic consequence. Finally, functional modules were manually curated from annotation categories. The KEGG enrichment analysis provides complementary support for selected metabolic, nucleotide and amino-acid components of the response-shift signal, but it does not independently validate every manually curated module category, including the broader redox/stress and envelope-associated summaries. These modules should therefore be interpreted as coarse biological summaries rather than experimentally validated pathways. Overall, the findings should be viewed as a prioritized, hypothesis-generating framework for future experimental validation.

Together, these findings support an interpretation in which vancomycin adaptation in *S. aureus* is accompanied by medium-associated remodeling of inducible response states rather than a uniform drug-response program. The study provides several concrete resources for follow-up work: a RefSeq locus-tag audit trail for all prioritized candidates, a transparent candidate-selection and evidence-ranking workflow, a conservative GSE254530 cross-dataset support table, retained candidate-associated RNase III-CLASH edge summaries with partner-response annotations, module-level response-rewiring statistics with KEGG pathway checks and the revision analysis scripts. Biologically, these resources converge on a focused set of hypotheses rather than a single validated resistance determinant: pyrR showed the clearest conservative cross-dataset differential-expression support; carbamoyl-P synthase, dihydroorotase and glnA represented top-ranked candidate-associated CLASH hotspots; and the strongest genome-wide and module-level response shifts were concentrated in the RPMI + 10% LB EVO-WT contrast. This integrated map is therefore intended as a reproducible, hypothesis-generating framework for prioritizing RNA-associated and metabolic response states for future experimental testing in *S. aureus* vancomycin adaptation.

## Data Availability

The datasets analyzed in this study are publicly available in the Gene Expression Omnibus (GEO). The discovery transcriptomic dataset is available under accession number GSE149213. The independent vancomycin-response RNA-seq dataset used for conservative cross-dataset assessment is available under accession number GSE254530. The RNase III-CLASH RNA-RNA interaction dataset is available under accession number GSE158830. All candidate-level summaries, RefSeq annotation audit information, candidate workflow tables, retained CLASH edge summaries, CLASH partner response annotations, module definitions, medium-interaction result tables, KEGG pathway-enrichment outputs and contrast-level result tables generated during this study are provided in the Supplementary material. Analysis scripts, R session information and the output manifest are provided as Supplementary code.
